# Association of Pesticides and Kidney Function among Adults in the US Population 2001–2010

**DOI:** 10.3390/ijerph181910249

**Published:** 2021-09-29

**Authors:** En-Tzu Wan, Darsy Darssan, Shamshad Karatela, Simon A. Reid, Nicholas John Osborne

**Affiliations:** 1 School of Public Health, The University of Queensland, Brisbane 4006, Australia; entzu.wan@uq.net.au (E.-T.W.); d.darssan@uq.edu.au (D.D.); s.karatela@uq.edu.au (S.K.); simon.reid@uq.edu.au (S.A.R.); 2 Department of Strategy Planning, Far Eastern Memorial Hospital, Banqiao District, New Taipei City 22060, Taiwan; 3 Australian Institute of Tropical Health and Medicine (AITHM), James Cook University, Townsville 4870, Australia; 4 European Centre for Environment and Human Health, University of Exeter, Truro TR1 3HD, UK; 5 School of Population Health, University of NSW, Kensington 2052, Australia

**Keywords:** pesticides, organophosphate insecticide, malathion, kidney function, NHANES

## Abstract

Chronic kidney disease of unknown cause is prevalent in a range of communities; however, its etiology remains unclear. We examined the association between pesticide exposures and the risk of kidney function loss using four waves of the National Health and Nutrition Examination Survey (NHANES) to identify a pathological pathway. We pooled data from four cross-sectional waves of NHANES, with 41,847 participants in total. Exposure to malathion increased the risk of low kidney function (aOR = 1.26, 95% CI = 1.01–1.56) in the adjusted model. Increased risk of low kidney function was not found among those exposed to 2,4-D (aOR = 0.88, 95% CI = 0.72–1.09), 3,5,6-trichloropyridinol (aOR = 0.96, 95% CI = 0.83–1.12), and 3-PBA (aOR = 1.03, 95% CI = 0.94–1.13). Our findings provide evidence of altered kidney function in people exposed to malathion, highlighting the potential of organophosphate pesticides’ role in renal injury.

## 1. Introduction

Chronic kidney disease (CKD) is a condition of permanent nephron damage and renal function loss [1]. Classic risk factors of CKD, such as hypertension (HTN) and diabetes (DM), account for most of the CKD burden in high-income countries [1]. However, numerous cases of CKD have emerged with unknown etiology (CKDu, definition of exclusion, i.e., no HTN nor DM or other) in tropical countries, as most of the affected patients are asymptomatic or have mild symptoms of elevated serum creatinine levels, low-grade or no proteinuria, or chronic interstitial nephritis with variable glomerulosclerosis [2,3].

Chronic kidney disease with unknown cause (CKDu) has recently been reported in people from poor communities in Mesoamerica [4], Sri Lanka [5,6], and India [7,8]. Initially, CKDu was suspected to be an occupational disease associated with exposures in the agricultural workplace, such as heat stress and dehydration [4], pesticide spraying [9], heavy metals and agrochemicals [10], or the use of NSAIDs [11]. Nonagricultural workers and other community members could also be at risk of CKDu, as environmental contamination from heavy metals and pesticide residues [10,12] or use of folk medicines containing heavy metals or aristolochic acid [13] may be contributing to the disease. Spraying pesticides without personal protective equipment (PPE) or working with contaminated soil have been suggested as likely exposure pathways for pesticides [3].

Organophosphate and the pyrethroid group of insecticides are the most commonly used pesticides in agricultural and domestic settings. Multiple adverse effects of pesticide exposure on kidney function and structure have been identified in animal studies. Mesnage et al. (2015) demonstrated the nephrotoxicity of pesticides, which triggered epigenetic effects and pathophysiological changes in kidney function [14]. In addition, pyrethroid exposure in rats causes oxidative stress that induces tissue damage [15], whilst chlorpyrifos accumulates in adipose tissues and in the liver and kidney, where it disrupts plasma membranes, leading to tissue damage and loss of enzyme activity [16].

Several epidemiological studies have been conducted to explore chronic pesticide exposures leading to kidney function loss in human beings. A prospective study of people in the USA found that long-term exposure to herbicides, such as paraquat, increased the risk of end-stage renal disease (ESRD) among commercial pesticide applicators (adjusted HR = 2.15, 95% CI = 1.11–4.15 [17]. However, this study was not able to recognize the mechanism leading to ESRD because it did not take pesticide components into account. Studies conducted in Sri Lanka had conflicting results due to the high disparity of study designs [18]. Although a self-report questionnaire is the main approach to assess pesticide exposures and other information, misclassification of participants and the lack of strategies to consider confounding factors vary the findings [19,20].

The etiology of CKDu remains unclear with the current evidence provided by studies. The objective of this study was to examine the association between pesticide exposure and the risk of kidney function loss using four waves of the National Health and Nutrition Examination Survey (NHANES) to elucidate a potential pathological pathway. The NHANES is a program of studies that aims to assess the health and nutritional status of adults and children in the United States [21]. It uses a multistage, probability sampling design to select nationally representative civilian and noninstitutionalized US residents [21]. Data from individuals who participated in the NHANES study are collected through physical examination, computer-based questionnaires, and laboratory analyses of biomarkers, such as cholesterol level, creatinine level, C-reactive protein, and environmental exposures to heavy metals and pesticides from blood and urine samples.

## 2. Materials and Methods

### 2.1. Study Population

We pooled data from four independent NHANES study waves (2001–2002, 2003–2004, 2007–2008, and 2009–2010). The NHANES 2005–06 study was not included because measurements of several pesticides of our interest were not available in this study period.

We included every participant from the NHANES 2001–2004 and 2007–2010. Several pesticides were selected for analysis, representing pesticide classes of organophosphate, phenoxy, and pyrethroid and also included the known nephrotoxin cadmium. We conducted several subpopulation analyses for 2,4-dichlorophenoxyacetic acid (2,4-D), 3,5,6-trichloropyridinol, 3-phenoxybenzoic acid (3-PBA), and malathion by only including participants from aged 20 to 80, as well as those who did not have missing pesticide and serum creatinine measurements. Participants over 80 years old were excluded in order to reduce biases from older adults who were more likely to have been institutionalized. All participants provided written informed consent during recruitment as part of the NHANES study protocol [21].

### 2.2. Kidney Function Data

Serum creatinine concentration was available for each participant in the NHANES. We calculated the estimated glomerular filtration rate (eGFR) based on the Chronic Kidney Disease–Epidemiology Collaboration (CKD–EPI) equation [22] (Equation (1): example for White or other male shown here; Black/White, male/female equations used in calculations).

eGFR = 141 × min (Scr/κ, 1) α × max (Scr/κ, 1)^−1.209^ × 0.993 Age × (1.018 if female) × (1.159 if Black) mL/min/1.73 m^2^
(*κ* is 0.7 if female, 0.9 if male, Scr is serum creatinine (mg/dL) (1)

Kidney function was classified into five stages on the basis of the eGFR level: more than 90 mL/min per 1.73 m^2^ (stage 1), 60–89 mL/min per 1.73 m^2^ (stage 2), 30–59 mL/min per 1.73 m^2^ (stage 3), 15–29 mL/min per 1.73 m^2^ (stage 4), and fewer than 15 mL/min per 1.73 m^2^ (stage 5) [1]. Participants in this study were considered to have CKD if their eGFR level was in stage 3 or stage 4. Participants in this study were considered to have “poor kidney function” if their eGFR level was in stage 3 or stage 4 (those corresponding to stage 5 CKD were excluded due to either small sample size or being unable to determine if these persons were undertaking dialysis) [23].

### 2.3. Measurement of Pesticides

The NHANES study measured various types of pesticide metabolite in urine specimens from a one-third subsample or participants that were randomly selected from the total participants in each wave. Analysis method details are documented at the NHANES website (https://wwwn.cdc.gov/nchs/nhanes/ContinuousNhanes (accessed on 1 August 2021)). Data on the concentration of the following herbicide and pesticides or their metabolites were available for analysis: 2,4-D herbicide, organophosphate insecticides, 3,5,6-trichloropyridinol (chlorpyrifos metabolite), malathion, and pyrethroid insecticide metabolite 3-PBA. The target analytes were extracted and concentrated from the urine matrix using an automated solid-phase extraction system, and there was no major change of laboratory methodology in different NHANES waves [21].

The limit of detection (LOD) varied among pesticide metabolites and heavy metals across each study wave. Therefore, we applied the maximum limit of detection (LODmax) to distinguish between nondetectable and detectable measurements from the laboratory. We assigned a substitution to all pesticides and heavy metal levels below the LODmax with the value of LODmax divided by the square root of two [24].

### 2.4. Other Variables

The following data were available from the NHANES dataset: age, sex, race/ethnicity, poverty–income ratio (PIR, a proxy of household socioeconomic status), smoking, and alcohol consumption behaviors and were collected via self-reported questionnaires. PIR is a ratio of self-reported family income to the appropriate poverty threshold of a family, and this index is comparable across NHANES waves. A PIR of less than 1.0 indicates those below the official poverty threshold, and PIR values of 1.00 or greater represent people above the poverty threshold.

People who reported smoking at least 100 cigarettes during their lifetime and who, at the time they participated in the survey, reported smoking every day or some days from the self-report questionnaire were defined as current smokers. Former smokers are those who reported smoking at least 100 cigarettes during their lifetime but were not smoking at the time of participating in the survey. Current drinkers are those who self-reported drinking at least 12 alcoholic drinks in the year preceding the study questionnaire; abstinence was recorded if there were fewer than 12 alcoholic drinks in that year.

The health status of each participant was measured directly using the waist circumference (WC), systolic blood pressure (SBP), diastolic blood pressure (DBP), and fasting blood glucose levels. Hypertension was defined according to the JNC 7 report as systolic blood pressure (SBP) greater than 140 mmHg, diastolic blood pressure (DBP) greater than 90 mmHg, or having any history of taking antihypertensive medications [25]. Diabetes was defined according to the definition of the American Diabetes Association (ADA) as either a fasting glucose level greater than 126 mg/dL or having any history of taking diabetic medications to decrease blood sugar levels [26]. Data on the cadmium concentration determined from blood samples in the NHANES is also included in this study as a positive control for the pesticide exposure.

### 2.5. Statistical Analysis

Sampling weights were used along with primary sampling units (PSUs) and strata. Stratification and clustering provided in the NHANES study were used in all statistical analyses to account for the complex, multistage sampling study design to represent the noninstitutionalized US population, as well as to obtain accurate estimates [21]. Following the NHANES analytical guidelines [27], the new sampling weight for the pooled NHANES data was calculated by dividing the 2-year weights for each period by 4 via Stata command (svyset) before analysis. In this study, the 8-year MEC weight was used to analyze the demographic characteristics of all participants. Pesticide subsample weights of 2,4-D, 3,5,6-trichloropyridinol, malathion, and 3-PBA, respectively, were used to take account for analyses that were conducted from a smaller subsample in each wave.

We described demographic variables using weighted means, standard deviation, and a 95% confidence interval (CI), as well as weighted percentage. Log transformation for pesticides and cadmium levels were applied in multivariate analyses. We utilized logistic regression to estimate the odds ratios (OR) and 95% CIs of the association between log-pesticide level and kidney function. OR estimation of log cadmium with poor kidney function was included as a positive control, as cadmium is a nephrotoxic metal presenting in various pesticides that leads to direct exposure to workers and the community through contaminated soil and water. Covariates including age, sex, race/ethnicity, PIR (continuous variable), smoking, and the NHANES waves were adjusted in every multivariate regression model.

By excluding participants with previous hypertension and diabetes history, we also performed a sensitivity analysis to assess the robustness of findings and assess a potential pathological pathway for underdetermined CKD [28].

Original NHANES data available online were converted to the Stata file format for management prior to data analysis. All statistical analyses were conducted using Stata version 15.1 (College Station, TX, USA). The Taylor series linearization method was applied to account for stratification and clustering.. Ethics for the collection of the original NHANES data and consent for participation were addressed by the NHANES Ethics Review Board (ERB) (see https://www.cdc.gov/nchs/nhanes/irba98.htm (accessed on 1 August 2021)).

## 3. Results

### 3.1. Demographic Characteristics of the Study Population

A total of 41,847 participants were included in the NHANES 2001–2004 and 2007–2010 (Table 1). Fifty one percent (51.1%) of the participants were females and the weighted mean age was 36.0 years old (SD: 0.26). The majority of participants (67.7%) were non-Hispanic White, followed by non-Hispanic Black (12.0%), Mexican American (9.1%), and other races (6.3% of multiracial people and 5.0% of other Hispanic). For the socioeconomic status of this study population, 15.9% of the population lived under the threshold of poverty (PIR < 1).

A total of 5.1% of the participants (1776 people) had CKD according to the definition described in Section 3.2. Just under half of the participants were either current smokers (23.3%) or former smokers (24.6%). Symptoms of hypertension and diabetes were present in 29.9% and 18.5% of the participants, respectively.

### 3.2. Risk of Low Kidney Function from Pesticide Exposures

The odds ratio of low kidney function risk was presented after pesticide concentrations were log transformed (Table 2). The odds of low kidney function were higher in the malathion subgroup, as malathion increased the risk of low kidney function among them in the adjusted model (aOR = 1.26, 95% CI = 1.01–1.56). A significantly increased risk of low kidney function was not found among 2,4-D, 3,5,6-trichloropyridinol (chlorpyrifos), and 3-PBA.

### 3.3. Sensitivity Analysis

In the sensitivity analysis, we excluded participants with previous hypertension and diabetes history (Table 3). Results were mostly similar to the main analyses.

## 4. Discussion

We examined if exposure to pesticides was associated with kidney dysfunction, such as CKD and acute kidney injury, while adjusting for a range of risk factors. By using an extant cohort with a range of exposure and outcome measures, we were also able to examine subgroups of kidney dysfunction that mimic CKDu, i.e., kidney dysfunction without diabetes and hypertension. In this study, we observed an increased risk of low kidney function associated with malathion exposure. The association between malathion and low kidney function was also present when excluding participants that had diabetes or hypertension, suggesting an association between malathion and CKDu is possible. We did not find that 2,4-D, chlorpyrifos, and 3-PBA exposures were associated with the risk of low kidney function in the NHANES 2001–2004 and 2007–2010 population. The increased risk of low kidney function was in the same effect size as our positive control, the known nephrotoxin cadmium.

We investigated the association of exposure to four pesticides from different classes and kidney function in a representative sample of the US noninstitutionalized population using four NHANES waves. Measures of pesticide exposure (and cadmium) were derived from biosampling, using a range of measurement techniques giving a known concentration of toxin exposure for individuals (https://www.cdc.gov/nchs/nhanes/index.htm (accessed on 1 August 2021)). In order to make the study generalizable to the US population, the study was designed using strategies including unequal probability sampling, nonresponse adjustment, and post-stratification adjustment [26].

The cross-sectional study design is the key limitation of the NHANES and this study. A cross-sectional design is prone to reverse causation, preventing any conclusions on the direction of the association between pesticide exposures and kidney function. In addition, only single measurements of pesticides were determined, which does not represent chronic exposure. Similarly, kidney dysfunction is only measured at one time point, as opposed to a more classical CKD measure (two measures, three months or longer apart). The limitations of using the NHANES data highlight the need for longitudinal studies that will aid in establishing the temporality and directionality of the relationship between exposure and outcome. Although a small number of individuals in this cohort may be taking medication that may reduce kidney function, this is thought to not influence the relationship with pesticide exposure. Similarly, routes of exposure of pesticide can only be speculated at with this dataset but may include oral (via pesticide residues on food) or inhalation (via exposure on application, drift after spraying, or exposure in domestic or occupational buildings that have been sprayed).

Animal studies have identified herbicides and pesticides as causes of tissue damage and renal dysfunction. An animal study found a significant increase in plasma urea level and creatinine among rats exposed to 2,4-D for 28 consecutive days [28], and increased urea and creatinine level after acute chlorpyrifos exposures [16]. In a study in rats, exposure to malathion was found to increase kidney weight, as well as serum creatinine and urea levels [29], which are signals of kidney injury and kidney function loss.

Several studies in humans have suggested pesticides, including malathion, are linked to nephrotoxicity [30]. An El Salvadoran study did not find a relationship between “ever use” of 2,4-D, organophosphates, and pyrethroids and reduced kidney function among sugarcane workers after harvest season based on self-reported exposure [4]. Another study in Nicaragua also did not find an association between “ever use” of 2,4-D or chlorpyrifos and low kidney function [31]. The authors identified the lack of biomarker data and the lack of longitudinal design as key limitations to accurately quantifying the level of pesticide exposure [31]. Another weakness was the use of a self-report questionnaire, which may lead to information bias as the accuracy of what workers recall may be different if they are surveyed during or after harvest season.

More recent studies using data from a prospective cohort study did not find a positive association between cumulative lifetime use of 2,4-D, chlorpyrifos, and malathion exposure and ESRD among licensed pesticide applicators in the US [17]. This is important because the authors quantified the level of lifetime pesticide exposure by collecting the intensity and duration of pesticide use using a self-administered questionnaire [17].

Malathion is the most widely used OP in the US [32] and is used in agricultural settings as well as for eradicating ectoparasites or household insects, as it is relatively less toxic than other OPs [33]. Malathion exposure is found to affect kidney function in acute intoxication, as evidenced by a case of acute renal insufficiency with proteinuria [34] and a case of acute kidney injury and nephrotic syndrome after malathion inhalation for 15 days [35]. However, epidemiological studies have not elucidated the relationship between malathion exposure and kidney function loss/CKD [17], potentially due to dose or memory recall of pesticides used.

While little is known about the nephrotoxic pathological pathways of pesticides with most evidence derived from animal studies [29], the pathological pathway will depend on the class of toxin involved. Organophosphates have been implicated in nephrotoxic activity in animals via oxidative stress (reduced glutathione metabolism) [30] and in porcine cell culture via oxidative stress at the tubular level [31].

## 5. Conclusions

This study provides evidence that exposure to OP malathion increases the risk of low kidney function in the general US population. There is a need to explore the interaction between pesticide exposure (acute and chronic) and other CKD risk factors, such as diabetes, blood pressure, heat stress, and environmental toxins. Further research is required to investigate if other pesticides are associated with kidney function loss in longitudinal studies, and in a range of exposure settings.

## Figures and Tables

**Table 1:** Demographic characteristics of participants in the National Health and Nutrition Examination Survey conducted in the US between 2001–2004 and 2007–2010.

Characteristics ^†^	All Participants (*N* = 41,847) ^1^	2,4-D (*n* = 6232) ^2^	3,5,6 (*n* = 4994) ^3^	3-PBA (*n* = 4910) ^4^	Malathion (*n* = 3557) ^5^
**Sex, % (95% CI)**					
Male	48.9 (48.4–49.4)	48.7 (47.3–50.1)	48.6 (46.9–50.3)	48.4 (46.7–50.1)	48.6 (46.5–50.7)
Female	51.1 (50.6–51.7)	51.3 (50.0–52.8)	51.4 (49.7–53.1)	51.7 (49.9–53.3)	51.4 (49.4–53.5)
Age (years), mean (SD, 95% CI), *n* = 41,847	36.0 (0.26, 35.5–36.6)	45.7 (0.27, 45.1–46.2)	45.9 (0.33, 45.2–46.5)	46.0 (0.38, 45.3–46.6)	46.8 (0.36, 46.0–47.5)
Waist circumference (cm), mean (SD, 95% CI), *n* = 34,964	90.2 (0.21, 89.8–90.6)	97.3 (0.26, 96.7–97.8)	97.3 (0.31, 96.7–98.0)	97.2 (0.31, 96.6–97.8)	98.0 (0.34, 97.3–98.7)
Serum creatinine (mg/dL), mean (SD, 95% CI), *n* = 19,728	0.87 (0.01, 0.86–0.87)	0.87 (0.01, 0.86–0.88)	0.87 (0.01, 0.86–0.88)	0.87 (0.01, 0.86–0.89)	0.87 (0.01, 0.86–0.89)
**Race, % (*n*, 95% CI)**					
Mexican American	9.1 (9836, 7.4–11.1)	8.3 (1218, 6.7–10.2)	8.2 (956, 6.5–10.3)	8.3 (950, 6.6–10.4)	8.7 (646, 6.4–11.8)
Other Hispanic	5.0 (3192, 3.8–6.6)	4.9 (495, 3.7–6.5)	5.1 (447, 3.6–7.1)	5.0 (435, 3.6–7.1)	4.9 (385, 3.4–7.1)
Non-Hispanic White	67.7 (17,274, 64.1–71.0)	69.8 (3071, 66.2–73.1)	69.8 (2439, 65.6–73.6)	70.1 (2407, 66.0–73.8)	69.0 (1703, 63.5–73.9)
Non-Hispanic Black	12.0 (9512, 10.3–13.9)	10.7 (1149, 9.1–12.7)	10.6 (917, 8.8–12.7)	10.4 (890, 8.7–12.5)	10.6 (643, 8.7–12.9)
Other Race/multiracial	6.3 (2033, 5.4–7.4)	6.4 (299, 5.4–7.6)	6.3 (235, 5.2–7.7)	6.2 (228, 5.1–7.5)	6.8 (180, 5.4–8.6)
**Family poverty-income ratio (PIR), % (*n*, 95% CI)**					
Below poverty (PIR < 1)	15.9 (10,365, 14.8–17.0)	12.6 (1122, 11.5–13.7)	12.8 (898, 11.7–14.0)	12.7 (876, 11.6–13.8)	13.3 (684, 11.9–14.8)
At or above poverty (PIR ≥ 1)	84.1 (31,482, 83.0–85.2)	87.4 (5110, 86.3–88.5)	87.2 (4096, 86.0–88.3)	87.3 (4034, 86.2–88.5)	86.7 (2873, 85.2–88.1)
**Cigarette smoking, % (*n*, 95% CI), *n* = 22,576** ^6^					
Current smoker	23.3 (4967, 22.2–24.4)	23.4 (1423, 21.9–24.8)	23.0 (1128, 21.4–24.9)	23.0 (1098, 21.3–24.8)	21.6 (777, 19.5–23.8)
Former smoker	24.6 (5791, 23.5–25.6)	24.2 (1578, 23.0–25.5)	24.1 (1258, 22.6–25.6)	24.3 (1248, 22.9–25.9)	23.7 (874, 22.2–25.4)
Nonsmoker	52.2 (11,818, 50.6–53.7)	52.4 (3226, 50.7–54.1)	52.9 (2604, 50.7–55.0)	52.7 (2560, 50.5–54.8)	54.7 (1903, 52.0–57.4)
**Alcohol consumption, % (*n*, 95% CI), *n* = 41,831** ^7^					
Current drinker	9.7 (2993, 8.9–10.6)	12.3 (856, 11.2–13.4)	11.4 (661, 10.2–12.7)	11.5 (654, 10.2–12.8)	10.8 (457, 9.2–12.6)
Abstinence	90.3 (38,838, 89.4–91.2)	87.7 (5373, 86.6–88.8)	88.6 (4330, 87.3–89.8)	88.5 (4253, 87.2–89.8)	89.2 (3097, 87.4–90.8)
**Hypertension, % (*n*, 95% CI), *n* = 29,382** ^8^					
Yes	29.9 (9026, 28.4–31.4)	34.9 (2394, 33.1–36.7)	34.7 (1941, 32.8–36.7)	34.7 (1910, 32.8–36.8)	35.2 (1428, 33.0–37.5)
No	70.2 (20,356, 68.7–71.6)	65.1 (3443, 63.3–66.9)	65.3 (2790, 63.4–67.2)	65.3 (2740, 63.3–67.2)	64.8 (1976, 62.5–67.0)
**Diabetes, % (*n*, 95% CI), *n* = 14,946** ^9^					
Yes	18.5 (3331, 17.6–19.5)	19.2 (855, 17.5–20.9)	19.9 (723, 18.0–21.8)	19.8 (711, 17.9–21.8)	22.9 (587, 20.4–25.6)
No	81.5 (11,615, 80.5–82.4)	80.9 (2600, 79.1–82.5)	80.1 (2103, 78.2–82.0)	80.2 (2067, 78.2–82.1)	77.1 (1473, 74.4–79.6)
**Chronic kidney disease, % (*n*, 95% CI), *n* = 26,619**					
Yes	5.1 (1776, 94.3–95.4)	4.8 (423, 4.2–5.4)	4.9 (346, 4.3–5.6)	5.0 (346, 4.5–5.7)	5.3 (267, 4.7–6.1)
No	94.9 (24,843, 4.6–5.7)	95.2 (5809, 94.6–95.8)	95.1 (4648, 94.4–95.7)	95.0 (4564, 94.3–95.6)	94.7 (3290, 93.9–95.4)

^†^ N presented in the first column is all of the participants who had non-missing values.^1^ Every participant in the NHANES 2001–2004 and 2007–2010 is included. MEC weight applied. ^2^ 2,4-D is 2,4-dichlorophenoxyacetic acid. Only participants aged 20 to 80 and with 2,4-D data available are included. Pesticide weight applied. ^3^ 3,5,6 is 3,5,6-trichloropyridinol. Only participants aged 20 to 80 and with 3,5,6-trichloropyridinol data available are included. Pesticide weight applied. ^4^ 3-PBA is 3-phenoxybenzoic acid. Only participants aged 20 to 80 and with 3-PBA data available are included. Pesticide weight applied. ^5^ Only participants aged 20 to 80 and with malathion data available are included. Pesticide weight applied. ^6^ (1) Current smoker: smoked at least 100 cigarettes during lifetime and report smoking every day or some days at the time of participating in the survey. (2) Former smoker: smoked at least 100 cigarettes during lifetime and report not smoking at the time of participating in the survey. (3) Nonsmoker: smoked fewer than 100 cigarettes during lifetime. (4) Missing value: either refuse, don’t know, or missed answering the two questions. ^7^ (1) Current drinker: self-reported drinking at least 12 alcoholic drinks in the year preceding the study questionnaire. (2) Abstinence: self-reported drinking less than 12 alcoholic drinks in the year preceding the study questionnaire. ^8^ Either systolic blood pressure (SBP) greater than 140 mmHg, or diastolic blood pressure (DBP) greater than 90 mmHg, or the use of antihypertensive medications. ^9^ Either fasting glucose level greater than 126 mg/dL or the use of diabetic medications to decrease blood sugar level.

**Table 2:** Weighted logistic regression of pesticide exposures and low kidney function for participants in the National Health and Nutrition Examination Survey conducted in the US between 2001–2004 and 2007–2010.

Pesticide (*n* Samples)	Crude OR	Adjusted OR *
log cadmium (*n* = 19,468)	1.40 (1.30–1.50)	1.21 (1.05–1.38)
log 2,4-D (*n* = 6232)	1.05 (0.89–1.23)	0.88 (0.72–1.09)
log 3,5,6-trichloropyridinol (*n* = 4994)	1.07 (0.95–1.20)	0.96 (0.83–1.12)
log malathion diacid (*n* = 3557)	1.30 (1.15–1.46)	1.26 (1.01–1.56)
log 3-phenoxybenzoic acid (*n* = 4910)	0.97 (0.88–1.06)	1.03 (0.94–1.13)

* Adjusted for age, sex, poverty–income ratio, ethnicity, smoking, and NHANES wave.

**Table 3:** Sensitivity analysis of pesticide and low kidney function after excluding participants with hypertension and diabetes in the National Health and Nutrition Examination Survey conducted in the US between 2001–2004 and 2007–2010.

Pesticide (*n* Samples)	Crude OR	Adjusted OR *
log cadmium (*n* = 17,508)	1.48 (1.36–1.61)	1.30 (1.10–1.52)
log 2,4-D (*n* = 5620)	1.12 (0.93–1.34)	0.97 (0.76–1.25)
log 3,5,6-trichloropyridinol (*n* = 4480)	1.08 (0.93–1.25)	0.93 (0.79–1.10)
log malathion diacid (*n* = 3140)	1.34 (1.16–1.54)	1.32 (1.01–1.73)
log 3-phenoxybenzoic acid (*n* = 4405)	0.96 (0.88–1.05)	1.02 (0.93–1.12)

* Adjusted for age, sex, poverty–income ratio, ethnicity, smoking, and NHANES wave.

## Data Availability

All data used in this study are freely available at https://www.cdc.gov/nchs/nhanes/index.htm (accessed on 1 August 2021).

## References

[1] Levey A.S., Coresh J. (2012). Chronic kidney disease. Lancet.

[2] Nanayakkara S., Komiya T., Ratnatunga N., Senevirathna S.T., Harada K.H., Hitomi T., Gobe G., Muso E., Abeysekera T., Koizumi A. (2012). Tubulointerstitial damage as the major pathological lesion in endemic chronic kidney disease among farmers in North Central Province of Sri Lanka. Environ. Health Prev. Med..

[3] Jayasumana C., Orantes C., Herrera R., Almaguer M., Lopez L., Silva L.C., Ordunez P., Siribaddana S., Gunatilake S., De Broe M.E. (2017). Chronic interstitial nephritis in agricultural communities: A worldwide epidemic with social, occupational and environmental determinants. Nephrol. Dial. Transplant..

[4] Garcia–Trabanino R., Jarquin E., Wesseling C., Johnson R.J., Gonzalez–Quiroz M., Weiss I., Glaser J., Vindell J.J., Stockfelt L., Roncal C. (2015). Heat stress, dehydration, and kidney function in sugarcane cutters in El Salvador––A cross–shift study of workers at risk of Mesoamerican nephropathy. Environ. Res..

[5] Chandrajith R., Nanayakkara S., Itai K., Aturaliya T.N., Dissanayake C.B., Abeysekera T., Harada K., Watanabe T., Koizumi A. (2011). Chronic kidney diseases of uncertain etiology (CKDue) in Sri Lanka: Geographic distribution and environmental implications. Environ. Geochem. Health.

[6] Jayasumana C., Paranagama P., Agampodi S., Wijewardane C., Gunatilake S., Siribaddana S. (2015). Drinking well water and occupational exposure to Herbicides is associated with chronic kidney disease, in Padavi–Sripura, Sri Lanka. Environ. Health.

[7] Ganguli A. (2016). Uddanam Nephropathy/Regional Nephropathy in India: Preliminary Findings and a Plea for Further Research. Am. J. Kidney Dis..

[8] John O., Gummudi B., Jha A., Gopalakrishnan N., Kalra O., Kaur P., Kher V., Kumar V., Machiraju R., Ookalkar D. (2021). Chronic kidney disease of unknown aetiology In India: What do we know and where do we need to go. Kid. Int. Rep..

[9] Rajapakse S., Shivanthan M.C., Selvarajah M. (2016). Chronic kidney disease of unknown etiology in Sri Lanka. Int. J. Occup. Environ. Health.

[10] Soderland P., Lovekar S., Weiner D.E., Brooks D.R., Kaufman J.S. (2010). Chronic kidney disease associated with environmental toxins and exposures. Adv. Chronic Kidney Dis..

[11] Gooch K., Culleton B.F., Manns B.J., Zhang J., Alfonso H., Tonelli M., Frank C., Klarenbach S., Hemmelgarn B.R. (2007). NSAID Use and Progression of Chronic Kidney Disease. Am. J. Med..

[12] Agampodi S.B., Amarasinghe G.S., Naotunna P., Jayasumana C.S., Siribaddana S.H. (2018). Early renal damage among children living in the region of highest burden of chronic kidney disease of unknown etiology (CKDu) in Sri Lanka. BMC Nephrol..

[13] Debelle F.D., Vanherweghem J.-L., Nortier J.L. (2008). Aristolochic acid nephropathy: A worldwide problem. Kidney Int..

[14] Mesnage R., Arno M., Costanzo M., Malatesta M., Seralini G.E., Antoniou M.N. (2015). Transcriptome profile analysis reflects rat liver and kidney damage following chronic ultra–low dose Roundup exposure. Environ. Health.

[15] Nasuti C., Cantalamessa F., Falcioni G., Gabbianelli R. (2003). Different effects of Type I and Type II pyrethroids on erythrocyte plasma membrane properties and enzymatic activity in rats. Toxicology.

[16] Tanvir E.M., Afroz R., Chowdhury M., Gan S.H., Karim N., Islam M.N., Khalil M.I. (2016). A model of chlorpyrifos distribution and its biochemical effects on the liver and kidneys of rats. Hum. Exp. Toxicol..

[17] Lebov J.F., Engel L.S., Richardson D., Hogan S.L., Hoppin J.A., Sandler D.P. (2016). Pesticide use and risk of end–stage renal disease among licensed pesticide applicators in the Agricultural Health Study. Occup. Environ. Med..

[18] World Health Organization (2016). International Expert Consultation on Chronic Kidney Disease of Unknown Etiology.

[19] Wanigasuriya K.P., Peiris–John R.J., Wickremasinghe R., Hittarage A. (2007). Chronic renal failure in North Central Province of Sri Lanka: An environmentally induced disease. Trans. R. Soc. Trop. Med. Hyg..

[20] Athuraliya N.T., Abeysekera T.D., Amerasinghe P.H., Kumarasiri R., Bandara P., Karunaratne U., Milton A.H., Jones A.L. (2011). Uncertain etiologies of proteinuric–chronic kidney disease in rural Sri Lanka. Kidney Int..

[21] Zipf G., Chiappa M., Porter K.S., Ostchega Y., Lewis B.G., Dostal J. (2013). National Health and Nutrition Examination Survey: Plan and Operations, 1999–2010.

[22] Levey A.S., Stevens L.A., Schmid C.H., Zhang Y.L., Castro A.F., Feldman H.I., Kusek J.W., Eggers P., Van Lente F., Greene T. (2009). A new equation to estimate glomerular filtration rate. Ann. Intern. Med..

[23] Barr D.B., Allen R., Olsson A.O., Bravo R., Caltabiano L.M., Montesano A., Nguyen J., Udunka S., Walden D., Walker R.D. (2005). Concentrations of selective metabolites of organophosphorus pesticides in the United States population. Environ. Res..

[24] Chobanian A.V., Bakris G.L., Black H.R., Cushman W.C., Green L.A., Izzo J.L., Jones D.W., Materson B.J., Oparil S., Wright J.T. (2003). The Seventh Report of the Joint National Committee on Prevention, Detection, Evaluation, and Treatment of High Blood Pressure: The JNC 7 report. JAMA.

[25] Resnick H.E., Harris M.I., Brock D.B., Harris T.B. (2000). American Diabetes Association diabetes diagnostic criteria, advancing age, and cardiovascular disease risk profiles: Results from the Third National Health and Nutrition Examination Survey. Diabetes Care.

[26] Johnson C.L., Paulose–Ram R., Ogden C.L., Carroll M.D., Kruszon–Moran D., Dohrmann S.M., Curtin L.R. (2013). National Health and Nutrition Examination Survey: Analytic Guidelines, 1999–2010.

[27] Pearce N., Caplin B. (2019). Let’s take the heat out of the CKDu debate: More evidence is needed. Occup. Environ. Med..

[28] Tayeb W., Nakbi A., Trabelsi M., Miled A., Hammami M. (2012). Biochemical and histological evaluation of kidney damage after sub–acute exposure to 2,4–dichlorophenoxyacetic herbicide in rats: Involvement of oxidative stress. Toxicol. Mech. Methods.

[29] Selmi S., Rtibi K., Grami D., Sebai H., Marzouki L. (2018). Malathion, an organophosphate insecticide, provokes metabolic, histopathologic and molecular disorders in liver and kidney in prepubertal male mice. Toxicol. Rep..

[30] Badr A.M. (2020). Organophosphate toxicity: Updates of malathion potential toxic effects in mammals and potential treatments. Environ. Sci. Pollut. Res. Int..

[31] Wesseling C., Aragon A., Gonzalez M., Weiss I., Glaser J., Rivard C.J., Roncal–Jimenez C., Correa–Rotter R., Johnson R.J. (2016). Heat stress, hydration and uric acid: A cross–sectional study in workers of three occupations in a hotspot of Mesoamerican nephropathy in Nicaragua. BMJ Open.

[32] Bonner M.R., Coble J., Blair A., Beane Freeman L.E., Hoppin J.A., Sandler D.P., Alavanja M.C.R. (2007). Malathion Exposure and the Incidence of Cancer in the Agricultural Health Study. Am. J. Epidemiol..

[33] Bogen K.T., Singhal A. (2017). Malathion dermal permeability in relation to dermal load: Assessment by physiologically based pharmacokinetic modeling of in vivo human data. J. Environ. Sci. Health Part B.

[34] Albright R.K., Kram B.W., White R.P. (1983). Malathion Exposure Associated With Acute Renal Failure. JAMA.

[35] Yokota K., Fukuda M., Katafuchi R., Okamoto T. (2017). Nephrotic syndrome and acute kidney injury induced by malathion toxicity. BMJ Case Rep..

